# Don’t worry about the anchor-item setting in longitudinal learning diagnostic assessments

**DOI:** 10.3389/fpsyg.2023.1112463

**Published:** 2023-02-09

**Authors:** Xinyue Yu, Peida Zhan, Qipeng Chen

**Affiliations:** ^1^School of Psychology, Zhejiang Normal University, Jinhua, China, Jinhua, China; ^2^Key Laboratory of Intelligent Education Technology and Application of Zhejiang Province, Jinhua, China

**Keywords:** learning diagnosis, longitudinal assessment, anchor-item design, diagnostic classification model, DINA model

## Abstract

Previous longitudinal assessment experiences for multidimensional continuous latent constructs suggested that the set of anchor items should be proportionally representative of the total test forms in content and statistical characteristics and that they should be loaded on every domain in multidimensional tests. In such cases, the set of items containing the unit Q-matrix, which is the smallest unit representing the whole test, seems to be the natural choice for anchor items. Two simulation studies were conducted to verify the applicability of these existing insights to longitudinal learning diagnostic assessments (LDAs). The results mainly indicated that there is no effect on the classification accuracy regardless of the unit Q-matrix in the anchor items, and even not including the anchor items has no impact on the classification accuracy. The findings of this brief study may ease practitioners’ worries regarding anchor-item settings in the practice application of longitudinal LDAs.

## Introduction

In educational and psychological measurement, cognitive or learning diagnostic assessments (LDAs) aim to provide diagnostic feedback on the current state of student learning (Rupp et al., 2010). With the popularity of formative assessments, longitudinal LDAs, which evaluate students’ latent attributes (e.g., knowledge and skills) and identify their strengths and weaknesses over a period, have received attention from researchers in recent years. By utilizing longitudinal LDAs, researchers can describe students’ learning development trajectories and verify the effectiveness of remedial interventions (e.g., Chen et al., 2017; Zhan et al., 2019a; Tang and Zhan, 2021).

Several longitudinal diagnostic classification models (DCMs) have been proposed to provide methodological support for data analysis in longitudinal LDAs (for a review, see Zhan, 2020), such as the higher-order latent structural model-based models (e.g., Huang, 2017; Zhan et al., 2019a; Pan et al., 2020; Zhan and He, 2021) and the hidden Markov model (or latent transition analysis)-based models (e.g., Chen et al., 2017; Kaya and Leite, 2017; Madison and Bradshaw, 2018; Wang et al., 2018). Currently, however, as a fresh research topic, there are still some issues in longitudinal LDAs that have not been explored clearly, besides model development, hindering their practical application.

Past approaches to modeling latent construct change have been based on repeated measurement data from multiple administrations of the same test or parallel tests at different occasions. In practice, especially for high-stake educational assessments, the use of the same test at multiple occasions is not always feasible. In addition, perfectly parallel tests do not exist; thus, the variation in results on different occasions may be partly due to the non-parallelism error of the instruments, which is difficult to quantify. A more commonly observed practice of test administration over time involves different test forms that share a common set of items called anchor-items rather than using the same test repeatedly or parallel tests (Kolen and Brennan, 2004; Paek et al., 2014; Zhan et al., 2019a). Unfortunately, the anchor-item setting in longitudinal LDAs has not yet been systematically studied.

There have been a great number of studies on the comparability between raw scores and estimates of latent constructs on different occasions, both in classical test theory and item response theory (e.g., Embretson, 1991; Kolen and Brennan, 2004; von Davier et al., 2011). Typically, to establish a common scale, one must have a common set of anchor items that are shared across occasions. In longitudinal studies, because the same group of respondents took the test multiple times, the anchor items were set primarily to allow changes in observed scores to be attributed to changes in latent constructs rather than differences in item parameters on different occasions. Experience suggests that assessments should have at least 20% of the length of a total test to anchor the parameters to the common scale (Kolen and Brennan, 2004). However, since the latent constructs are treated as multidimensional discrete variables in LDAs, the applicability of those recommendations, mainly from tests for the unidimensional continuous latent construct, to longitudinal LDAs still needs to be explored.

Despite the lack of research, experience from multidimensional tests still gives us some insight into the anchor-item setting in longitudinal LDAs (Kolen and Brennan, 2004; Wang and Nydick, 2020). First, the set of anchor items is suggested to be proportionally representative of the total test forms in terms of content and statistical characteristics. Second, anchor items are suggested to be loaded on every domain in multidimensional tests. Third, to help ensure similar behavior, each anchor-item is suggested to occupy a similar location (item number) on different occasions.

Currently, in the field of longitudinal LDAs with anchor items, there are some studies that follow those insights (e.g., Zhan et al., 2019a) and some that do not (e.g., Zhan, 2020), but none of them specify the reasons for this or explore the impact of the anchor-item setting. For example, in Zhan et al. (2019a) studies, the first 20% of items on each occasion were set as anchor items, and each anchor item examined each attribute separately, namely, the correspondence between anchor items and attributes expressed by a unit Q-matrix^1^, such as $\left(\begin{array}{cc} 1 & 0\\ 0 & 1 \end{array}\right)$ for two attributes.

In LDAs, the set of items containing the unit Q-matrix is the smallest unit that can represent the whole test in terms of content and statistical characteristics. Previous research has found that the unit Q-matrix is critical for the completeness of the Q-matrix and the identifiability of the DCM (Gu and Xu, 2020). The purpose of constructing the Q-matrix is to achieve the complete differentiation of all latent classes in the latent variable (class) space, which is also the main purpose of LDA. And the unit-Q matrix is the necessary condition to achieve that purpose (Chiu, 2013; Gu and Xu, 2020; Wang et al., 2020). Therefore, in terms of achieving the main purpose of LDA, the set of items containing the unit Q-matrix seems to be the natural choice for anchor items.

However, a recent study of the hidden Markov model-based longitudinal DCM pointed out that no anchor items are necessary in longitudinal LDAs because the scale for DCMs in non-arbitrary (Madison and Bradshaw, 2018) or the interpretation of attribute is deterministic (Ma et al., 2021). The results of Madison and Bradshaw (2018) study indicated that even in tests without anchor items, the hidden Markov model-based longitudinal DCM can classify respondents accurately and reliably. However, since their study only explored the pre-test/post-test scenario (i.e., longitudinal assessments with two occasions), it is not clear whether their conclusion is applicable to LDAs with more occasions, and whether it is applicable to the higher-order latent structural model-based longitudinal DCMs.

Overall, although existing studies can provide some insights, the research on anchor-item settings in longitudinal LDAs is relatively lacking, which hinders the application of anchor-item design in longitudinal LDAs. This study focuses on three questions to explore the impact of anchor-item setting on diagnostic classification accuracy of the higher-order latent structural model-based longitudinal DCMs in longitudinal LDAs with more than two occasions. Whether it is necessary to set the items containing the unit Q-matrix as anchor items? if so, whether increasing the anchor-item ratio is beneficial to increase diagnostic classification accuracy? if not, whether it is necessary to set anchor items?

For simplicity and without loss of generality, the longitudinal higher-order deterministic-inputs noisy and gate (Long-DINA) model (Zhan et al., 2019a), which is a representative higher-order latent structural model-based longitudinal DCM, was used in this study. The rest of the paper starts with a brief review of the Long-DINA model, followed by two simulation studies to explore the impact of various anchor-item settings on classification accuracy in longitudinal LDA. Finally, the authors summarized the findings and discussed potential directions for future research.

## Brief review of longitudinal higher-order deterministic-inputs noisy and gate model

Let *y*_*nit*_ be the item response of person *n* (*n* = 1,…, *N*) to item *i* (*i* = 1,…, *I*) at occasion *t* (*t* = 1,…, *T*). The long-DINA model can be expressed as follows:

First order:


(1) $$\text{logit}\left(P\left(y_{\text{nit}}=1|\alpha_{nt},g_{it},s_{it}\right)\right)=\lambda_{0it}+\lambda_{1it}\overset{K}{\underset{k=1}{\prod }}\alpha_{\text{nkt}}^{q_{\text{ikt}}},$$

Second order:


(2) $$\text{logit}\left(P\left(\alpha_{\text{nkt}}=1|\theta_{n},\beta_{k},\delta_{k}\right)\right)=\beta_{k}\theta_{n}-\delta_{k},$$

Third order:


(3) $$\theta_{n}={\left(\theta_{n1},\ldots ,\theta_{nT}\right)}^{\prime }\sim MVN\left(\mu ,\Sigma \right),$$

where λ_0__*it*_ and λ_1__*it*_ are the intercept and interaction parameters for item *i* at occasion *t*, respectively; **α**_*nt*_ = (α*n*_*1t*_,…, α_*nKt*_)’ denotes person *n*’s attribute profile at occasion *t*, α_*nkt*_∈{0, 1}; *q*_*ikt*_ is the element in an *I*-by-*K* polytomous Q-matrix at occasion *t*; θ_*nt*_ is person *n*’s general ability at occasion *t*; β_*k*_ and δ_*k*_ are the slope and difficulty parameters of attribute *k* on all occasions, respectively, since the same latent structure is assumed to be invariance at different occasions; **μ** = (μ_1_,…, μ_*T*_)’ is the mean vector, and **Σ** is the variance–covariance matrix:


(4) $$\Sigma =\left[\begin{array}{ccc} \sigma_{1}^{2} & \; & \;\\ \vdots & \ddots & \;\\ \sigma_{T1} & \cdots & \sigma_{T}^{2} \end{array}\right]$$

where σ_1__*T*_ is the covariance of the first and *T*th general abilities. As a starting and reference point for subsequent occasions, θ_*n1*_ is constrained to follow a standard normal distribution, which is μ_1_ = 0 and $\sigma_{1}^{2}=1$. The mean values and variances of θ_*nt*_ (*t* ≥ 2) are free to estimate. When *T* = 1, the Long-DINA model is reduced to the higher-order DINA model for cross-sectional data analysis (de la Torre and Douglas, 2004).

## Simulation studies

Two simulation studies were conducted to explore the impact of various anchor-item settings on classification accuracy in longitudinal LDAs. Study 1 aims to explore whether setting the items containing the unit Q-matrix as anchor items would be beneficial in increasing diagnostic classification accuracy, while Study 2 aims to explore the influence of different anchor-item ratios on the diagnostic classification accuracy. Study 1 is the basis for Study 2, and we separated these two studies mainly to avoid the interaction between different operational variables, which may cause difficulties in the interpretation of the results.

### Simulation study 1

#### Design and data generation

For ease of expression, **Q** and **Q**_a_ were used to denote the Q-matrix of the whole test and that of the anchor items, respectively, and **R** was used to denote the unit Q-matrix. Three factors were manipulated. First, the number of items on each occasion was set at $I_{t}$= 20 and 30. Second, the sample size was set at *N* = 100 and 500. Third, five conditions were set for anchor-item settings (see Table 1). Also, the number of required attributes was fixed to *K* = 4, and the number of occasions was fixed to *T* = 3. A total of 100 data sets were generated in each simulated condition.

**Table 1:** Anchor-item settings in Study 1.

Condition	Number of anchor items	Number of R in anchor items	Anchor-item location
1	4	0	9 ~ 12
2	4	1	1 ~ 4
3	8	0	9 ~ 16
4	8	1	1 ~ 4 and 9 ~ 12
5	8	2	1 ~ 8

There are 20 or 30 items on each occasion; anchor items were located remained the same on three occasions.

On each occasion, the **Q** was generated as **Q** = (**R**, **R**, Q^*^)^T^, to ensure the identifiability of the DCM (Gu and Xu, 2020), where the **R** is a 4 × 4 unit Q-matrix and the Q^*^ was randomly combined from 11 possible attribute patterns that required more than one attribute^2^. Since anchor items were located at the same location on three occasions, when some items in Q^*^ were selected as anchor items, the ***q***_*it*_s of them on the subsequent occasions (*t* ≥ 2) were fixed to the ones on the first occasion, ***q***_*i1*_. The **Q** was regenerated in each replication; namely, 100 **Q**s were generated.

**Figure 1:**
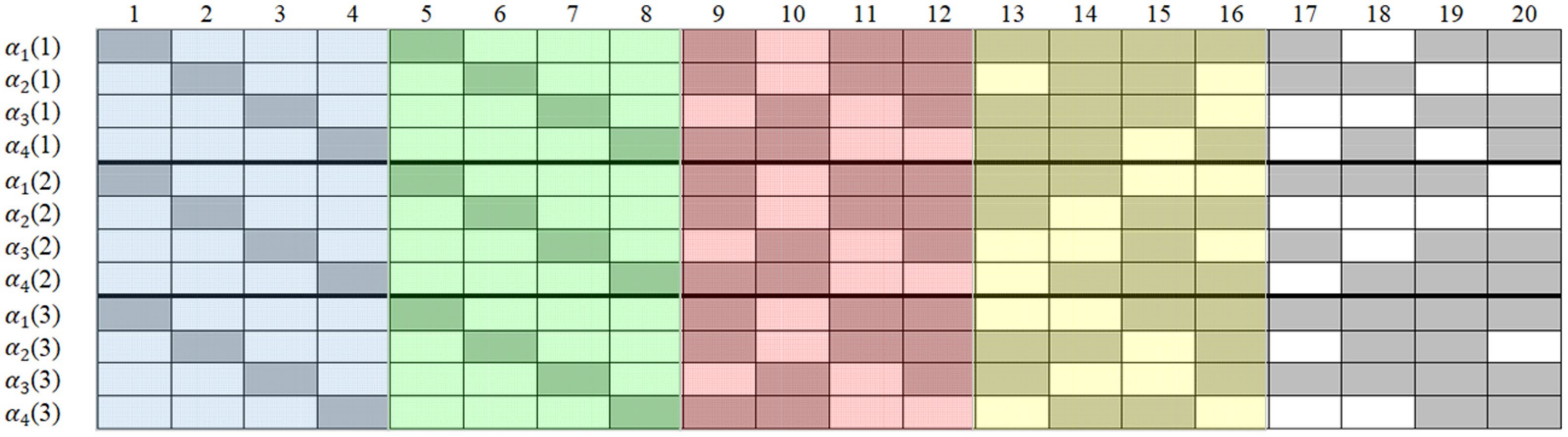
Sample Q-matrices with 20 items in Study 1. Gray means “1” and blank means “0”; occasion is in parentheses; simulated condition 1 contains anchor-items in the red area; simulated condition 2 contains anchor-items in the blue area; simulated condition 3 contains anchor-items in the red and yellow areas; simulated condition 4 contains anchor-items in the blue and red areas; simulated condition 5 contains anchor-items in the blue and green areas.

Five simulated conditions were considered (see Table 1): (1) four items outside **R** (i.e., in Q^*^) were designated as anchor items; (2) four items inside **R** were designated as anchor items; (3) eight items outside **R** were designated as anchor items; (4) four items inside and four items outside **R** were designated as anchor items; and (5) eight items inside two **R**s were designated as anchor items. Figure 1 displays the sample **Q**s with 20 items (the 30-item one just has 10 more items in the back). In the **Q** on each occasion, the first two 4 × 4 unit matrices were the two **R**s, and the remaining items in the Q^*^ were randomly generated and different in each replication. The different color areas and their combinations correspond to the five simulated conditions in Table 1. For example, the blue and red areas correspond to simulated condition 4. As already noted in the introduction, existing experience suggests that anchor items should occupy the same position at different item points, so to control for possible position effects, we fixed the anchor-item positions throughout the studies.

On each occasion, following the setting of Zhan et al. (2019b), item parameters were generated from a bivariate normal distribution with a negative correlation coefficient, as follows:


(5) $$\left(\begin{array}{c} \lambda_{0it}\\ \lambda_{1it} \end{array}\right)\sim N\left(\left(\begin{array}{c} -2.197\\ 4.394 \end{array}\right),\left(\begin{array}{cc} 1 & -0.6\\ -0.6 & 1 \end{array}\right)\right)$$

In such cases, there was a moderate negative correlation between the generated guessing (i.e., $\frac{\exp\left(\lambda_{0\text{it}}\right)}{1+\exp\left(\lambda_{0\text{it}}\right)}$ and slipping (i.e., 1-$\frac{\exp\left(\lambda_{0\text{it}}+\lambda_{1\text{it}}\right)}{1+\exp\left(\lambda_{0\text{it}}+\lambda_{1\text{it}}\right)}$) parameters; at this time, the probabilities of guessing and slipping presented a positively skewed distribution (average ≈ 0.1, minimum ≈ 0.01, and maximum ≈ 0.6), which was more realistic than fixing them to a specific value (Zhan et al., 2019b). When some items were selected as anchor items, the item parameters of those items on subsequent occasions (*t* ≥ 2) were fixed to those on the first occasion.

Additionally, for the latent structural parameters, $\delta_{k}$ were all set as 1.5 and $\beta ={\left(-1,-0.5,0.5,1\right)}^{\prime }$ for the four attributes. For general abilities, the correlations among them were set at 0.9. Between two consecutive occasions, the overall mean growth (i.e., $\mu_{t}-\mu_{t-1}$) was set as 0.5, and the overall scale change (i.e., $\frac{\sigma_{t}}{\sigma_{t-1}}$) was set as $\sqrt{1.25}$. The general abilities on *T* occasions were generated from a *T*-way multivariate normal distribution according to Eq. 3. On each occasion, the true attribute profile for each person was generated according to Eq. 2. Finally, the observed item responses were generated from Bernoulli ($p_{\text{nit}}$), where $p_{\text{nit}}$ was given in Eq. 1.

#### Analysis

The parameters of the Long-DINA model were estimated using full Bayesian estimation *via* the Markov Chain Monte Carlo algorithm, which was implemented in the JAGS software (Plummer, 2015). The JAGS code for the Long-DINA model can be found in Zhan et al. (2019c).

For each dataset, two Markov chains were used, and 10,000 iterations were run for each chain. The first 5,000 iterations in each chain were discarded as burn-ins. The remaining 5,000 iterations (each chain had 5,000 iterations) were run for the model parameter estimation. The potential scale reduction factor (PSRF; Brooks and Gelman, 1998) was computed to assess the convergence of every parameter. Values of PSRF less than 1.1 or 1.2 indicate convergence. Our results indicated that the PSRFs were almost less than 1.1, suggesting good convergence for the specified setting.

Also, the attribute correct classification rate (ACCR) and pattern correct classification rate (PCCR) were computed to evaluate the classification accuracy of attributes as ACCR=∑r=1Re∑n=1NI(α^nkr=αnkr)N×Re and PCCR=∑r=1Re∑n=1NI(α^nr=αnr)N×Re, where I(·) was an indicator function, α*_nkr_* and ${\hat{\alpha }}_{\text{nkr}}$ was the true and estimated status of person *n* for attribute *k* on occasion *r*, respectively; **α***_nr_* and ${\hat{\alpha }}_{nr}$ was the true and estimated attribute profile of person *n* on occasion *r*, respectively; *N* was the sample size, and *Re* was the number of replications. Following Zhan et al. (2019a), two kinds of PCCR were considered: PCCR on each occasion and longitudinal PCCR (LPCCR) for all occasions. The former focuses on whether *K* = 4 attributes on a given occasion can be correctly estimated, while the latter focuses on whether all *T* × *K* = 12 attributes can be correctly estimated.

#### Results

**Figure 2:**
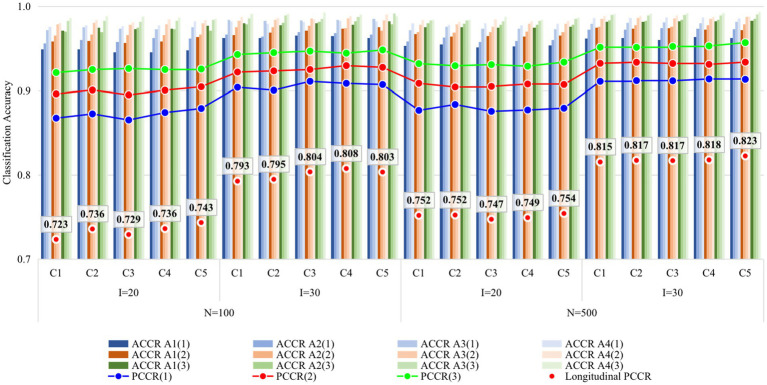
Recovery of attributes in Study 1. I, number of items; N, sample size; Occasion is in parentheses; C1, four items outside R were set as anchor items; C2, four items inside R were set as anchor items; C3, eight items outside R were set as anchor items; C4, four items inside and four items outside R were set as anchor items; C5, eight items inside two Rs were set as anchor items; R, unit Q-matrix. ACCR, attribute correct classification rate; PCCR, attribute pattern correct classification rate on each occasion (4 attributes); Longitudinal PCCR, PCCR on all three occasions (12 attributes).

**Figure 3:**
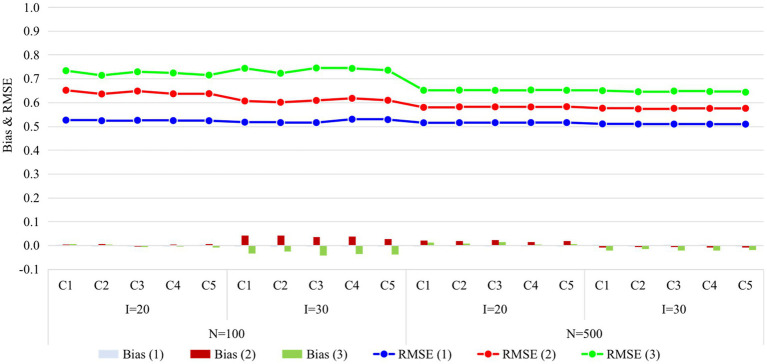
Recovery of ability in Study 1. I, number of items; N, sample size; Occasion is in parentheses; C1, four items outside R were set as anchor items; C2, four items inside R were set as anchor items; C3, eight items outside R were set as anchor items; C4, four items inside and four items outside R were set as anchor items; C5, eight items inside two Rs were set as anchor items; R, unit Q-matrix; RMSE, root mean square error.

Figures 2, 3 display the recovery of the attributes and the recovery of latent ability in Study 1, respectively. Except for some findings that are consistent with previous studies (e.g., classification accuracy increased with time, with more items, and with a larger sample size), the main focus of this study was on the performance differences of the five conditions. Obviously, the recovery of attributes and ability do not vary greatly under different conditions, especially when the sample size increased from 100 to 500, the consistency of the results under all conditions was higher. The results of Study 1 answered the first question of this study, that is, it seems that there is no need to set the items containing the unit Q-matrix as anchor items.

### Simulation study 2

#### Design, data generation, and analysis

Five ratios of anchor items were considered: 0, 20, 40, 60, and 80% (see Tables 2, 3). For the last four conditions, items containing one **R** were used as anchor items in all four conditions, and the number of items outside **R** was increased to operate the simulated conditions to avoid the influence of the number of unit Q-matrices. The data generation process and the data analysis process were consistent with those in Simulation Study 1.

**Table 2:** Anchor-item settings for conditions with 20 items in Study 2.

Condition	Ratio of anchor items (%)	Number of anchor items	Anchor-item location
1	0	0	–
2	20	4	1 ~ 4
3	40	8	1 ~ 4 and 9 ~ 12
4	60	12	1 ~ 4 and 9 ~ 16
5	80	16	1 ~ 4 and 9 ~ 20

Anchor items were located remained the same on three occasions; anchor items contain a unit Q-matrix (i.e., items 1 ~ 4) under all conditions, except condition 1.

**Table 3:** Anchor-item settings for conditions with 30 items in Study 2.

Condition	Ratio of anchor items (%)	Number of anchor items	Anchor-item location
1	0	0	-
2	20	6	1 ~ 4 and 9 ~ 10
3	40	12	1 ~ 4 and 9 ~ 16
4	60	18	1 ~ 4 and 9 ~ 22
5	80	24	1 ~ 4 and 9 ~ 28

Anchor items were located remained the same on three occasions; anchor items contain a unit Q-matrix (i.e., items 1 ~ 4) under all conditions, except condition 1.

#### Results

**Figure 4:**
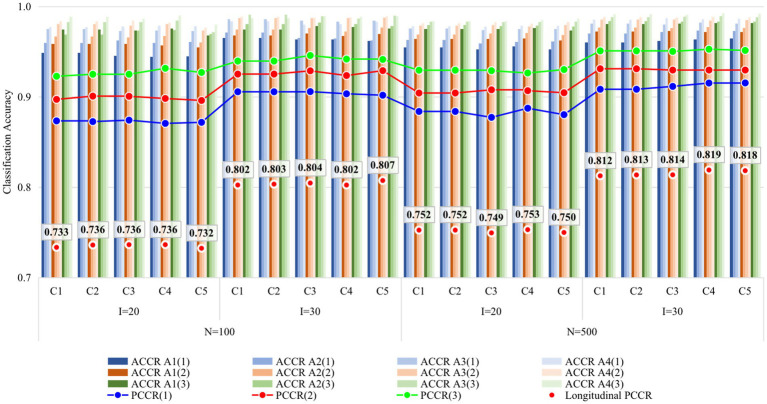
Recovery of attributes in Study 2. I, number of items; N, sample size; ACCR, attribute correct classification rate; PCCR, attribute pattern correct classification rate on each occasion (four attributes); LPCCR, PCCR on all three occasions (12 attributes); Occasion is in parentheses; C1, ratio of anchor items is 0%; C2, ratio of anchor items is 20%; C3, ratio of anchor items is 40%; C4, ratio of anchor items is 60%; C5, ratio of anchor items is 80%.

**Figure 5:**
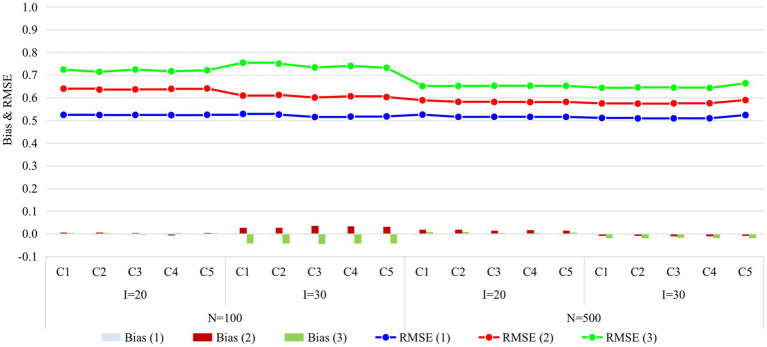
Recovery of ability in Study 2. I, number of items; N, sample size; Occasion is in parentheses; C1, ratio of anchor items is 0%; C2, ratio of anchor items is 20%; C3, ratio of anchor items is 40%; C4, ratio of anchor items is 60%; C5, ratio of anchor items is 80%; RMSE, root mean square error.

Figures 4, 5 display the recovery of the attributes and the recovery of latent ability in Study 2, respectively. Obviously, the classification accuracy and recovery of latent ability were quite similar across all conditions, indicating that simply increasing the ratio of anchor items outside the unit Q-matrix does not contribute substantially to classification accuracy. It is worth noting that even without any anchor items, the classification accuracy and recovery of latent ability were basically consistent with other conditions. The results of Study 2 answered the last two questions of this study, that is, different anchor-item ratios did not affect classification accuracy, and also, not having anchor items had no effect on classification accuracy.

## Conclusion and discussion

Previous longitudinal assessment experiences for multidimensional continuous latent constructs suggested that the set of anchor items should be proportionally representative of the total test forms in content and statistical characteristics, and that they should be loaded on every domain in multidimensional tests. In such cases, the set of items containing the unit Q-matrix, which is the smallest unit representing the whole test, seems to be the natural choice for anchor items. To verify the applicability of these existing insights to longitudinal LDAs, two simulation studies were conducted. The results mainly indicated that there is no effect on the classification accuracy regardless of the unit Q-matrix in the anchor items, and even not including the anchor items has no effect on the classification accuracy. The findings of this study may ease practitioners’ worries regarding anchor-item setting in the practice application of longitudinal LDAs.

The results of this brief study support Madison and Bradshaw (2018) view that no anchor items are necessary in longitudinal LDAs. This view applies not only to the hidden Markov model-based longitudinal DCM but also to the higher-order latent structural model-based longitudinal DCM. In addition, the findings of this study may ease practitioners’ worries regarding anchor-item setting in upcoming practice applications; namely, practice applicants do not seem to be overly worried about how anchor items are set in longitudinal LDAs, and even the absence of anchor items does not affect the accuracy of classifying respondents.

While this brief study focused on longitudinal assessments with three time points, the conclusions are applicable to longitudinal assessments with more than three time points as well. Supplementary Figure S1 depicts the attribute recovery in the simulated conditions with four time points of the two typical anchor item settings (no anchor item and four items inside **R**). The results indicated that the classification accuracy for both anchor settings remained high consistent across varied simulated conditions.

Previous researches which conducted in CTT and IRT have found that a certain percentage of anchor items are needed; however, in LDA, the attributes on different occasions are on the same scale because the interpretation of attribute is deterministic (Madison and Bradshaw, 2018; Ma et al., 2021). This essential difference leads to attribute estimates at different occasions that are naturally on a same scale, without the need to build linking between them through anchor items. Of course, due to the unobservability of latent variables, we cannot determine in advance whether the analyzed latent variables satisfy the binary certainty assumption. In recent years, some researchers have started to pay attention to the problem of non-determinism or continuity of attributes (e.g., Zhan et al., 2018; Ma et al., 2022). A more interesting direction is to explore how model misspecification and particularly the violation of binary certainty assumption of attributes will affect the use of anchor items.

Although the findings of this study are valuable in guiding how to set anchor items in the practical application of longitudinal LDA, the current study only considered some simple cases and still left some issues for further discussion. First, this study explores the performance of only one longitudinal DCM (i.e., the Long-DINA model) under different anchor item settings. Whether the findings would apply equally to other longitudinal DCMs [e.g., generalized DINA (de la Torre, 2011)-based longitudinal model] warrants further study. Second, only the commonly used internal anchor items, in which the score on anchor items contributes to the respondents’ total score on the test, were considered in this study. Whether the findings apply to the longitudinal LDA with external anchor items is still worth further study in the future. Third, the simulated conditions in this study follow some insights from experience (Kolen and Brennan, 2004), such as each anchor item being fixed at the same location on different occasions and anchor items being loaded on every domain (i.e., attributes), and did not explore the impact of anchor item setting under conditions that violate these insights. Fourth, to focus on the research topic, this brief report ignored the effect of some factors on classification accuracy, such as item quality, attribute hierarchy, misspecified Q-matrix, and local dependence between the responses to anchor-items. Fifth, herein, all item parameters of the same item were assumed to be invariant over time. However, item parameter drift might be expected for anchor items, and in that case, more work would be required to address this issue.

## Data availability statement

The raw data supporting the conclusions of this article will be made available by the authors, without undue reservation.

## Author contributions

XY conducted simulation studies and wrote the first draft. PZ provided the idea and revised the manuscript. QC assisted in the data analysis. All authors contributed to the article and approved the submitted version.

## Conflict of interest

The authors declare that the research was conducted in the absence of any commercial or financial relationships that could be construed as a potential conflict of interest.

## Publisher’s note

All claims expressed in this article are solely those of the authors and do not necessarily represent those of their affiliated organizations, or those of the publisher, the editors and the reviewers. Any product that may be evaluated in this article, or claim that may be made by its manufacturer, is not guaranteed or endorsed by the publisher.

## Footnotes

^1^Also known as the reachability matrix (Tatsuoka, 1986) for structurally independent attributes, which specifies the direct and indirect relationships among the attributes.

^2^Namely, (1100), (1010), (1001), (0110), (0101), (0011), (1110), (1101), (1011), (0111), and (1111).

## References

[ref1] Brooks S. P. Gelman A. (1998). General methods for monitoring convergence of iterative simulations. J. Comput. Graph. Stat. 7, 434–455. doi: 10.1039/b510875f

[ref2] Chen Y. Culpepper S. A. Wang S. Douglas J. (2017). A hidden Markov model for learning trajectories in cognitive diagnosis with application to spatial rotation skills. Appl. Psychol. Meas. 42, 5–23. doi: 10.1177/0146621617721250 29881110 PMC5978590

[ref3] Chiu C.-Y. (2013). Statistical refinement of the Q-matrix in cognitive diagnosis. Appl. Psychol. Meas. 37, 598–618. doi: 10.1177/0146621613488436

[ref4] de la Torre J. (2011). The generalized DINA model framework. Psychometrika 76, 179–199. doi: 10.1007/s11336-011-9207-7

[ref5] de la Torre J. Douglas J. (2004). Higher-order latent trait models for cognitive diagnosis. Psychometrika 69, 333–353. doi: 10.1007/BF02295640

[ref6] Embretson S. (1991). “Implications of a multidimensional latent trait model for measuring change,” in Best methods for the analysis of change: Recent advances, unanswered questions, future directions. eds. Collins L. M. Horn J. L. (Washington, DC: American Psychological Association), 184–197. doi: 10.1037/10099-012

[ref7] Gu Y. Xu G. (2020). Partial identifiability of restricted latent class models. Ann. Stat. 48, 2082–2107. doi: 10.1214/19-AOS1878

[ref8] Huang H. Y. (2017). Multilevel cognitive diagnosis models for assessing changes in latent attributes. J. Educ. Meas. 54, 440–480. doi: 10.1111/jedm.12156

[ref9] Kaya Y. Leite W. L. (2017). Assessing change in latent skills across time with longitudinal cognitive diagnosis modeling: an evaluation of model performance. Educ. Psychol. Meas. 77, 369–388. doi: 10.1177/0013164416659314, PMID: 29795918 PMC5965550

[ref10] Kolen M. J. Brennan R. L. (2004). Test equating, scaling, and linking: Methods and practices. New York, NY: Springer, doi: 10.1007/978-1-4757-4310-4

[ref11] Ma W. Chen J. Jiang Z. (2022). Attribute continuity in cognitive diagnosis models: impact on parameter estimation and its detection. Behaviormetrika 50, 217–240. doi: 10.1007/s41237-022-00174-y

[ref12] Ma W. Terzi R. de la Torre J. (2021). Detecting differential item functioning using multiple-group cognitive diagnosis models. Appl. Psychol. Meas. 45, 37–53. doi: 10.1177/0146621620965745, PMID: 33304020 PMC7711248

[ref13] Madison M. J. Bradshaw L. P. (2018). Assessing growth in a diagnostic classification model framework. Psychometrika 83, 963–990. doi: 10.1007/s11336-018-9638-5, PMID: 30264183

[ref14] Paek I. Park H. J. Cai L. Chi E. (2014). A comparison of three IRT approaches to examine ability change modeling in a single-group anchor test design. Educ. Psychol. Meas. 74, 659–676. doi: 10.1177/0013164413507062

[ref15] Pan Q. Qin L. Kingston N. (2020). Growth modeling in a diagnostic classification model (DCM) framework—a multivariate longitudinal diagnostic classification model. Front. Psychol. 11:1714. doi: 10.3389/fpsyg.2020.01714, PMID: 32903674 PMC7438873

[ref16] Plummer M. (2015). JAGS version 4.0.0 user manual. Available at: http://sourceforge.net/projects/mcmc-jags/

[ref17] Rupp A. Templin J. Henson R. (2010). Diagnostic assessment: Theory, methods, and applications. New York: Guilford.

[ref18] Tang F. Zhan P. (2021). Does diagnostic feedback promote learning? Evidence from a longitudinal cognitive diagnostic assessment. AERA Open 7, 10608–10615. doi: 10.1177/23328584211060804

[ref19] Tatsuoka M. M. (1986). Graph theory and its applications in educational research: a review and integration. Rev. Educ. Res. 56, 291–329. doi: 10.3102/00346543056003291

[ref20] von Davier M. Xu X. Carstensen C. H. (2011). Measuring growth in a longitudinal large-scale assessment with a general latent variable model. Psychometrika 76, 318–336. doi: 10.1007/s11336-011-9202-z

[ref21] Wang C. Nydick S. W. (2020). On longitudinal item response theory models: a didactic. J. Educ. Behav. Stat. 45, 339–368. doi: 10.3102/1076998619882026

[ref22] Wang W. Song L. Ding S. Wang T. Gao P. Xiong J. (2020). A semi-supervised learning method for Q-matrix specification under the DINA and DINO model with independent structure. Front. Psychol. 11:2120. doi: 10.3389/fpsyg.2020.02120, PMID: 33013538 PMC7511573

[ref23] Wang S. Yang Y. Culpepper S. A. Douglas J. A. (2018). Tracking skill acquisition with cognitive diagnosis models: a higher-order, hidden Markov model with covariates. J. Educ. Behav. Stat. 43, 57–87. doi: 10.3102/1076998617719727

[ref24] Zhan P. (2020). Longitudinal learning diagnosis: Minireview and future research directions. Front. Psychol. 11:1185. doi: 10.3389/fpsyg.2020.01185, PMID: 32719629 PMC7347960

[ref25] Zhan P. He K. (2021). A longitudinal diagnostic model with hierarchical learning trajectories. Educ. Meas. Issues Pract. 40, 18–30. doi: 10.1111/emip.12422

[ref26] Zhan P. Jiao H. Liao M. Bian Y. (2019a). Bayesian DINA modeling incorporating within-item characteristic dependency. Appl. Psychol. Meas. 43, 143–158. doi: 10.1177/0146621618781594, PMID: 30792561 PMC6376533

[ref27] Zhan P. Jiao H. Liao D. Li F. (2019b). A longitudinal higher-order diagnostic classification model. J. Educ. Behav. Stat. 44, 251–281. doi: 10.3102/1076998619827593

[ref28] Zhan P. Jiao H. Man K. Wang L. (2019c). Using JAGS for Bayesian cognitive diagnosis modeling: a tutorial. J. Educ. Behav. Stat. 44, 473–503. doi: 10.3102/1076998619826040

[ref29] Zhan P. Wang W.-C. Jiao H. Bian Y. (2018). Probabilistic-input, noisy conjunctive models for cognitive diagnosis. Front. Psychol. 9:997. doi: 10.3389/fpsyg.2018.00997, PMID: 29962994 PMC6010692

