# Supplementary material for: Don’t worry about the anchor-item setting in longitudinal learning diagnostic assessments
Source: Front Psychol. 2023 Feb 9;14:1112463. doi: 10.3389/fpsyg.2023.1112463 (PMC9948075; doi:10.3389/fpsyg.2023.1112463)
Supplement: Supplementary file 1 [file Data_Sheet_1.docx]

**Online Appendix**

***Don’t Worry About the Anchor-Item Setting in Longitudinal Learning Diagnostic Assessments***

**FIGURE A1**

*Sample Q-matrices with 20 items in Study 1.*

*Note:* Gray means “1” and blank means “0”; occasion is in parentheses; simulated condition 1 contains anchor-items in the red area; simulated condition 2 contains anchor-items in the blue area; simulated condition 3 contains anchor-items in the red and yellow areas; simulated condition 4 contains anchor-items in the blue and red areas; simulated condition 5 contains anchor-items in the red and green areas.

# FIGURE A2

# *Recovery of Ability in Study 1.*


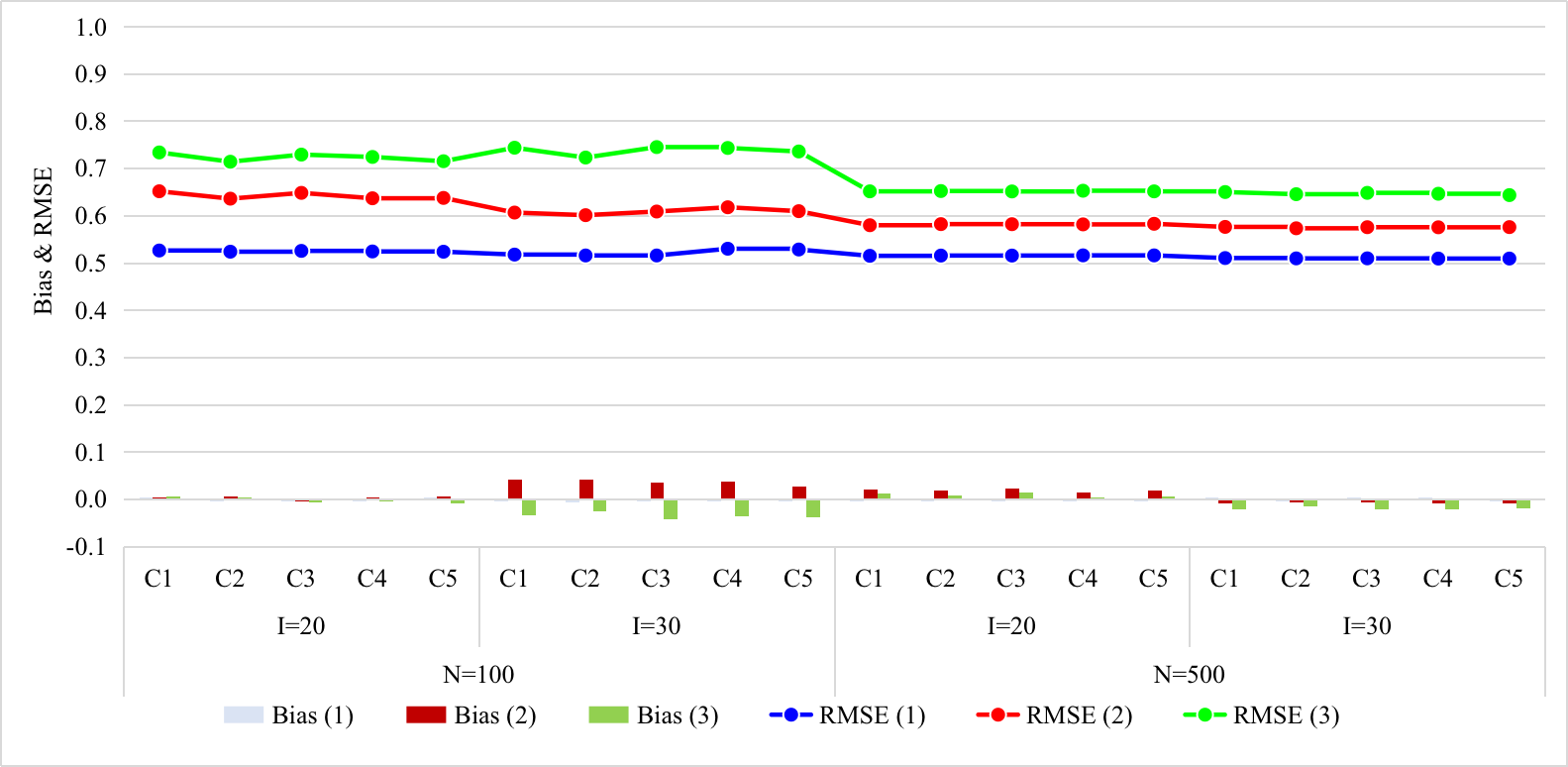


*Note*: I = number of items; N = sample size; ACCR = attribute correct classification rate; PCCR = attribute pattern correct classification rate on each occasion (4 attributes); Longitudinal PCCR = PCCR on all three occasions (12 attributes); Occasion is in parentheses; C1 = four items outside **R** were set as anchor items; C2 = four items inside **R** were set as anchor items; C3 = eight items outside **R** were set as anchor items; C4 = four items inside and four items outside **R** were set as anchor items; C5 = eight items inside two **R**s were set as anchor items; **R** = unit Q-matrix; RMSE = root mean square error.

# TABLE A1

*Anchor-item Settings for Conditions with 30 Items in Study 2.*

| Condition | Ratio of Anchor Items | Number of Anchor Items | Anchor-Item Location |
| --- | --- | --- | --- |
|  | 0% | 0 | - |
| 1 | 20% | 6 | 1~4 and 9~10 |
| 2 | 40% | 12 | 1~4 and 9~16 |
| 3 | 60% | 18 | 1~4 and 9~22 |
| 4 | 80% | 24 | 1~4 and 9~28 |

*Note*: Anchor items were located remained the same on three occasions; anchor items contain a unit Q-matrix (i.e., items 1~4) under all conditions, except condition 1.

# FIGURE A3

*Recovery of Ability in Study 2.*


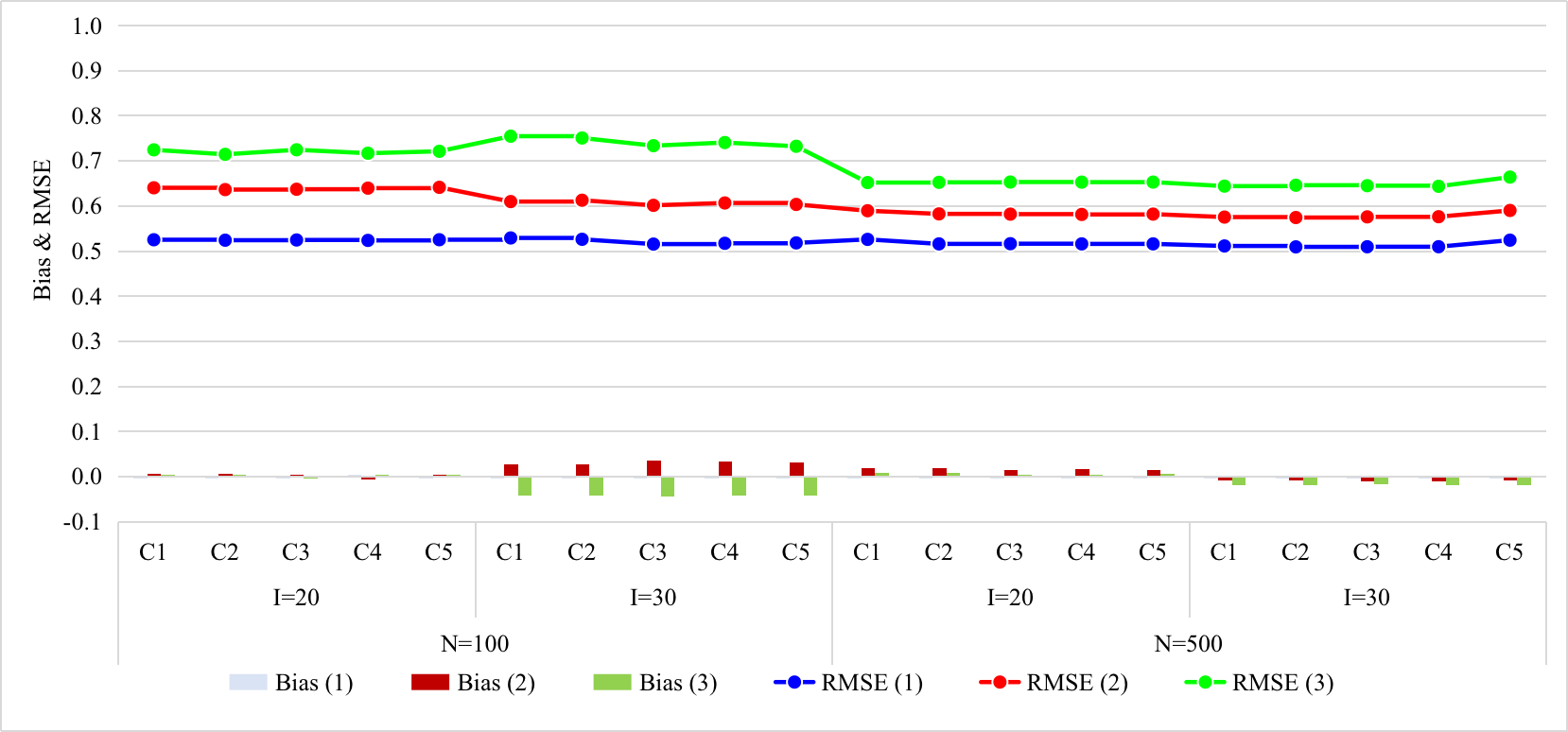


*Note*: I = number of items; N = sample size; ACCR = attribute correct classification rate; PCCR = attribute pattern correct classification rate on each occasion (four attributes); LPCCR = PCCR on all three occasions (12 attributes); Occasion is in parentheses; C1 = ratio of anchor items is 0%; C2 = ratio of anchor items is 20%; C3 = ratio of anchor items is 40%; C4 = ratio of anchor items is 60%; C5 = ratio of anchor items is 80%; RMSE = root mean square error.

# FIGURE A4

*Recovery of Attributes for Simulated Conditions with Four Time Points.*


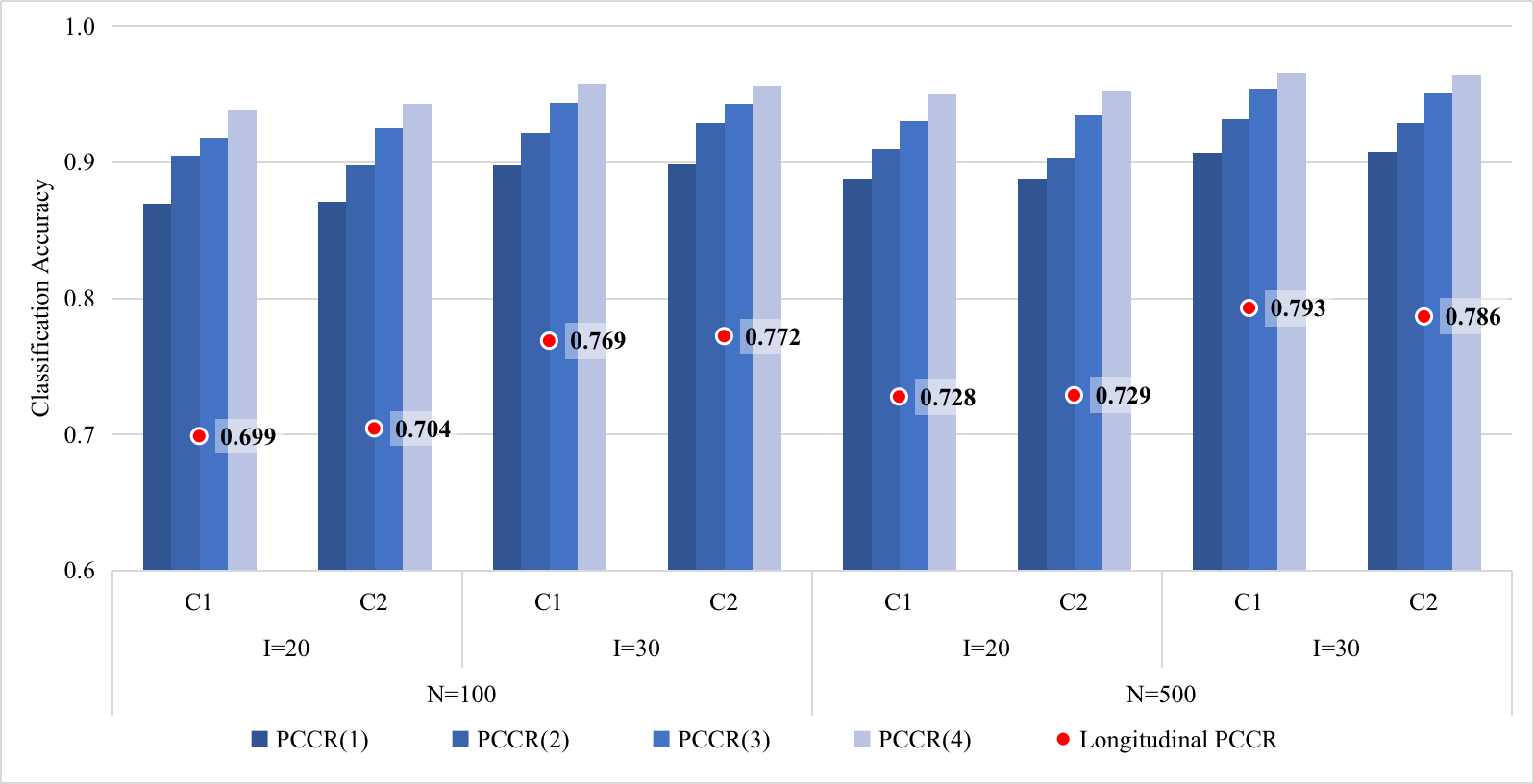


*Note*: I = number of items; N = sample size; PCCR = attribute pattern correct classification rate on each occasion (four attributes); LPCCR = PCCR on all three occasions (12 attributes); Occasion is in parentheses; C1 = no anchor items (i.e., ratio of anchor items is 0%); C2 = four items inside **R** were set as anchor items (i.e., ratio of anchor items is 20%).
